# Association between glutathione peroxidase 1 codon 198 variant and the occurrence of breast cancer in Rwanda

**DOI:** 10.1002/mgg3.367

**Published:** 2018-02-06

**Authors:** Thierry Habyarimana, Youssef Bakri, Pacifique Mugenzi, Jean Baptiste Mazarati, Mohammed Attaleb, Mohammed El Mzibri

**Affiliations:** ^1^ Biology and Medical Research Unit Centre National de l'Energie, des Sciences et des Techniques Nucléaires (CNESTEN) Rabat Morocco; ^2^ Biology of Human Pathologies Laboratory (BioPatH) Faculty of Science & Human Pathologies Center (GenoPatH) Mohammed V University Rabat Morocco; ^3^ Biomedical Services Department Rwanda Biomedical Center (RBC‐BIOS) Kigali Rwanda; ^4^ Rwanda Military Hospital (RMH) Kigali Rwanda; ^5^ King Faisal Hospital (KFH) Kigali Rwanda

**Keywords:** breast cancer, gene polymorphism, *GPX1*, reactive oxygen species, Rwanda

## Abstract

**Background:**

Glutathione peroxidase 1 gene (*GPX1*) is one of the antioxidant enzyme that remove the reactive oxygen species in a continuous process. Since the identification of a well‐characterized functional polymorphism named p.Pro198Leu (rs1050450 C>T) in *GPX1* gene, abundant studies have evaluated the association between p.Pro198Leu polymorphism and tumor risk in diverse population. But, the available results related to breast cancer are conflicting and absent in Africa. The present case–control study was planned to assess the presence of GPX1 Pro198Leu polymorphism in Rwanda population to determine whether it is associated with the risk of developing breast cancer.

**Methods:**

Genomic DNA from peripheral blood leukocytes of 41 patients with breast cancer and 42 healthy controls were enrolled and genotyped GPX1 Pro198Leu polymorphism by PCR amplification and DNA sequencing.

**Results:**

No significant difference in the frequencies of Pro/Pro (49%) and Pro/Leu (51%) genotypes in cancer cases and in controls (50% each) were found. The allelic frequencies of Pro and Leu were 74% versus 26% and 75% versus 25% in breast cancer cases and controls respectively. No association was observed in allele frequencies of Pro and Leu, and familial history. Only an overall association of GPX1 Pro198Leu with grade of cancer (Pro/Leu vs. Pro/Pro: *p *=* *.0200) was detected.

**Conclusion:**

The result of this study suggested that GPX1 Pro198Leu polymorphism could not be a risk factor for breast cancer in Rwanda. However, large‐scale studies on the effect of this polymorphism on the factors disturbing the redox homeostasis are needed for conclusive understanding.

## 1 INTRODUCTION

Breast cancer is a growing public health concern for many African nations and its incidence and mortality are increasing annually (Global Burden of Disease Cancer Collaboration, 2015). In Africa, breast cancer is commonly present at young ages and with advanced‐stage disease, mainly due to lack of screening programs, limited diagnostic capabilities, and inefficient treatment process (Adjei, Owusu‐Afriyie, Awuah, & Stalsberg, 2010; Bird, Hill, & Houssami, 2008; Farmer et al., 2010; Galukande, Wabinga, & Mirembe, 2015).

It's widely accepted that breast cancer in an heterogeneous disease and several risk factor are associated with breast cancer development, namely age, gender, ethnicity, past history of breast cancer, reproductive and hormonal factors, family history and genetic factors, exposure to ionizing radiation, and environmental and lifestyle factors (Jung et al., 2013). In this field, some endogenous factors (genomic variations) and/or exogenous factors (environmental exposures, lifestyle) can impact the balance of reactive oxygen species (ROS) leading to oxidative stress. Elevated levels of ROS and down regulation of ROS scavengers and/or antioxidant enzymes, are associated with initiation and progression of a number of human diseases and cancers including breast cancer (Gupta‐Elera, Garrett, Robison, & O'Neill, 2012; Halliwell, 2007; Lau, Wang, & Chiu, 2008; Loft et al., 2008).

Reactive oxygen species are highly reactive molecules that can damage DNA, proteins, and lipids and promote several carcinogenesis effects, such as increasing DNA mutation rate, deletions, gene amplification, rearrangements, and cell proliferation (Matés, Alonso, & Ma, 2012; Matés, Segura, & Alonso, 2008; Thannickal & Fanburg, 2000). In the organism, many genes are involved in ROS regulation (antioxidant enzymes, ROS scavengers) or production (mitochondrial genes in charge of the respiratory chain), affecting their function and efficiency (Bai, Leal, & Covarrubias, 2007; Wang et al., 2009). Among them, glutathione peroxidase 1 gene (*GPX1*,OMIM: 138320), encoding for an important antioxidant selenium‐dependent enzyme that catalyses the breakdown of hydrogen peroxide and organic hydroperoxides, resulting in the oxidation of glutathione (GSH) to glutathione disulphide (GSSG) (Brigelius‐Flohé, 1999; Kulak et al., 2013). *GPX1* has been reported to be implicated in oncogenesis and progression of several cancer types (Diwadkar‐Navsariwala & Diamond, 2004; Zhuo et al., 2009), it's overexpression suppresses intracellular ROS which attenuates growth factor receptor activation mediated by oxidative stress, resulting in decreased cellular proliferation (Handy et al., 2009; Oberley, 2005).

*GPX1* is located on chromosome position 3p21 and contains a genetic polymorphism (rs1050450) that results in either a proline (Pro) or leucine (Leu) at codon 198, described to be a risk factor for the development of various cancers, including lung cancer (Lee et al., 2006), prostate cancer, (Liwei, Wei, & Ruifa, 2012; Parlaktas, Atilgan, Gencten, Benli, & Ozyurt, 2015) and bladder cancer (Men, Zhang, Yang, & Shen, 2014; Paz‐y‐Mino et al., 2010).

In breast cancer, several population‐based studies have reported inconsistent results on the association between GPX1 Pro198Leu polymorphism and cancer risk. Some studies found that the leucine‐containing allele was more frequently associated with breast cancer than the proline‐containing allele while others findings reported no significant association (Cox, Hankinson, Kraft, & Hunter, 2004; Hu & Diamond, 2003). The meta‐analysis performed by Hu, Ning, and Wang (2010) covering three studies of Caucasian descent (Cox et al., 2004; Knight et al., 2004; Ravn‐Haren et al., 2006), one study of African descent (Hu & Diamond, 2003) and two studies of mixed ethnicity descent (Ahn et al., 2005; Cebrian et al., 2006) has not revealed any association between variant Leu allele and breast cancer susceptibility. However, in the subgroup analysis by ethnicity, no association was detected in Caucasians, while increased risk was found in Africans carrying variant Leu allele homozygote, deserving further investigation on large scale African populations. Therefore, we have planned to conduct this case–control study to assess the presence of GPX1 Pro198Leu polymorphism in Rwandan population to determine whether this polymorphism is associated with the risk of developing breast cancer in Rwandan patients.

## 2 MATERIAL AND METHODS

### 2.1 Ethical compliance

The protocol study was approved by the Rwanda National Ethics Committee (197/RNEC/2015) and informed consent was obtained from each participant.

### 2.2 Study population

A total of 41 breast cancer patients and 42 healthy women were enrolled in this study. All breast cancer cases were recruited in 2016 at Rwanda Military Hospital and King Faisal Hospital, both located at Kigali, Rwanda. From each breast cancer case, clinical and pathological data (age, tumor localization, histological subtype, tumor stage, and grade) were collected. Table 1 summarizes characteristics of the breast cancer patients and healthy individuals.

**Table 1:** Characteristics of the population description

	Patients (*n* = 41)	Controls (*n* = 42)
Age	40 (26–60)	25 (18–33)
Gender		
Females	40	39
Males	1	3
Cancer family history^a^	13	0
Histological subtypes		
Invasive ductal carcinoma	35	–
Unknown	6	–
Stage		
II	13	–
III	26	–
Unknown	2	–
Grade		
I	2	–
II	4	
III	34	–
Unknown	1	–

*n*, Number.

a Family history was defined as a first to second‐degree relative with any cancer type.

### 2.3 Blood sampling and DNA extraction

Approximately, 5 ml of the whole blood was taken by venepuncture into 5 ml vacutainers (Greiner Bio‐One, Germany) containing EDTA. Genomic DNA was isolated from all peripheral blood samples by using a commercial kit (Isolate II Genomic DNA Kit, BIOLINE) according to manufacturer's recommendations. DNA obtained was immediately used for PCR amplification or stored at −20°C until use.

### 2.4 *GPX1* Pro198Leu genotyping

p.Pro198Leu polymorphism screening was carried out by PCR amplification and DNA sequencing (Hadami et al., 2016). For PCR amplification primers were selected to flank the region of exon 2 containing the SNP rs1050450 C>T (NM_001329455.1); GPX1‐Ex2‐F primer (5′‐CGCCACCGCGCTTATGACCG‐3′) and GPX1‐Ex2‐R primer (5′‐GCAGCACTGCAACTGCCAAGCAG‐3′). PCR amplification was performed in a total volume of 25 μl, containing 1.5 mmol/L MgCl_2_, 200 μmol/L of each dNTP, 200 nmol/L of each primer, 0.25 U Platinum Taq DNA polymerase (Invitrogen), and 100 ng of genomic DNA in 1X PCR buffer. PCR reaction was performed as follows: After a first denaturation at 94°C for 7 min, follow 35 cycles of 94°C for 30 s, 60°C for 30 s, 72°C for 40 s. At the end of the last cycle, the mixtures were incubated at 72°C for 7 min. For every reaction, a negative control, in which DNA template was omitted from the amplification mixture, was included.

PCR products were purified using the Illustra ExoProStar 1‐Step enzymatic clean up system (GE Healthcare Life Sciences) and sequenced with forward strand on an ABI 3130XL DNA analyzer, using BigDye Terminator v3.1 Cycle Sequencing Kit (Applied Biosystems, Foster City, CA, USA).

Sequencing reactions were performed in a final volume of 10 μl, containing 1 μl of 2.5X Big Dye ready reaction mix v.3.1, 10 pmol of forward primer and 100 ng of purified PCR product. The mixtures were incubated at 96°C for 1 min and 25 cycles were performed, including denaturation at 96°C for 10 s, primer annealing at 50°C for 5 s and extension at 60°C for 4 min. The reactions were set to 30 μl. To eliminate the excess of labeled ddNTPs, sequencing reaction products were purified using Sephadex G‐50 gel‐exclusion chromatography (GE Healthcare Life Sciences). The sequences were analysed using Sequence Scanner v2.0 software (Applied Biosystems).

### 2.5 Statistical analysis

Statistical tests were performed using the OpenEpi software. Chi‐square test with Yates’ correction was used to evaluate the association of *GPX1* genotypes with the occurrence of bladder cancer and to examine the correlation between *GPX1* genotypes and cancer stage or grade. The statistical relationship was considered as significant if the derived *p*‐value was ≤.05. The estimated genotypic and allelic frequencies were associated with 95% confidence intervals calculated using the modified Wald test (Agresti‐Coull).

## 3 RESULTS

**Figure 1:**
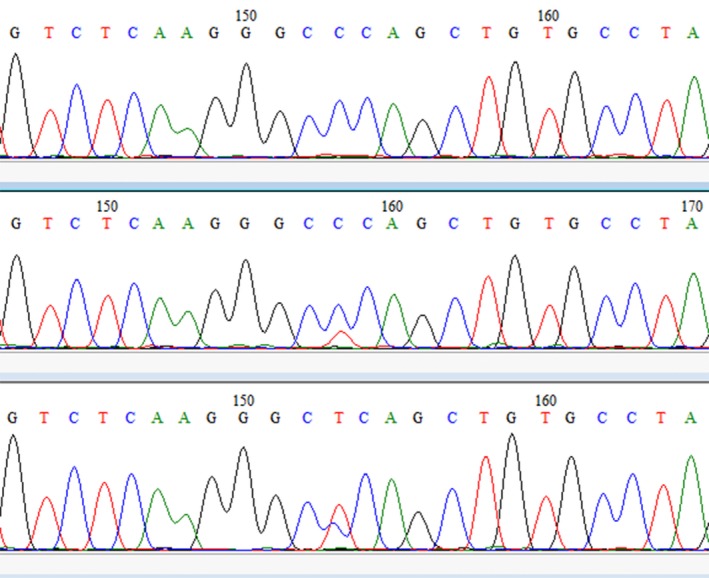
(a) A DNA sequence electrophoregram showing no C>T transition on both strands (CCC/CCC: Pro/Pro). (b) and (c) The DNA sequence chromatograms of C>T transition carries (CCC/CTC: Pro/Leu)

Amplification and direct sequencing of *GPX1* exon 2 for all 41 cases of BC and 42 controls were successfully conducted and revealed rs1050450 C>T substitution in exon2 resulting in p.Pro198Leu in both cancer specimens and controls. An example of obtained electrophoregrams is illustrated in Figure 1.

In our study, only two genotypes were detected (Pro/Pro and Pro/Leu). However, homozygous Leu genotype was found neither in cancer cases nor in controls. The genotype and allele frequencies of GPX1 Pro198Leu polymorphism are summarized in Table 2.

**Table 2:** Genotypic and allelic frequencies of GPX1 Pro198Leu polymorphism in patients and controls

Cases	*n*	Genotype			Allele	
		Pro/Pro % [95% CI]	Pro/Leu % [95% CI]	Leu/Leu % [95% CI]	Pro % [95% CI]	Leu % [95% CI]
Breast cancer	41	49 [39.42–58.65]	51 [41.35–60.58]	0 [0.0–4.441]	74 [64.58–81.64]	26 [18.36–35.42]
Controls	42	50 [40.38–59.62]	50 [40.38–59.62]	0 [0.0–4.441]	75 [65.65–82.5]	25 [17.5–34.35]
*P*		.500			.500	

rs1050450 C>T (NM_001329455.1).

CI, confidence intervals; *n*, number.

Overall, genotypic frequencies obtained in cancer cases were similar to that obtained in controls. Indeed, the frequencies of Pro/Pro and Pro/Leu genotypes were, respectively, 49% (20/41) and 51% (21/41) in cancer cases, and were 50% each in controls. Statistical analysis showed that no significant difference between cancer cases and healthy controls (*p *=* *.05).

Assessment of allelic frequencies showed that Pro allele was present in 74% of cases and 75% of controls, and Leu allele in 26% and 25% of cases and controls, respectively, and statistical analysis showed no significant difference between cases and controls.

The allelic frequencies of Pro and Leu were 74% versus 26% and 75% versus 25% in breast cancer cases and controls, respectively. No statistically significant difference allelic frequencies was observed of GPX1 Pro/Leu alleles between cancer and control groups (*p = *.05).

GPX1 Pro198Leu polymorphism was also evaluated according to familial history. As indicated in Table 3, both Pro/Pro and Pro/Leu genotype were detected in cancer cases and healthy controls with approximately similar frequencies. Moreover, allelic frequencies of Pro and Leu were 73.1% versus 26.9% and 75% versus 25% in breast cancer cases and controls respectively. Statistical analysis showed no significant difference between GPX1 Pro198Leu polymorphism and familial history of cancer (*p *>* *.05).

**Table 3:** Distribution of GPX1 Pro198Leu polymorphism according to familial history of breast cancer

Cases	*n*	Genotype		Allele	
		Pro/Pro % [95% CI]	Pro/Leu % [95% CI]	Pro% [95% CI]	Leu % [95% CI]
Cases with familial history	13	46.1 [36.66–55.83]	53.9 [44.17–63.34]	73.1 [63.63–80.86]	26.9 [19.14–36.37]
Sporadic cases	28	50 [40.38–59.62]	50 [40.38–59.62]	75 [65.65–82.5]	25 [17.5–34.35]
*p* a		.340		.442	

rs1050450 C>T (NM_001329455.1).

CI, confidence intervals; *n*, number.

a No case has the Leu/Leu genotype; therefore this genotype was not included in the statistical analysis.

GPX1 Pro198Leu polymorphism was also evaluated according to tumor grade and clinical stage and the results are reported in Table 4. According to cancer grade, the Pro/Pro and Pro/Leu genotypes were detected respectively in 38.5% and 61.5% of cases with clinical stage II, whereas in stage III, Pro/Pro was detected in 53.9% and Pro/Leu in 46.1% (2/6) of cancer cases. Statistical analysis showed a significant association between p.Pro198Leu polymorphism and breast cancer (*p *=* *.02).

**Table 4:** Distribution of GPX1 Pro198Leu polymorphism according to clinical stage and tumor grade

Cases	*n*	Genotype		*p* a	Allele		*p*
		Pro/Pro % [95% CI]	Pro/Leu % [95% CI]		Pro % [95% CI]	Leu % [95% CI]	
Clinical stage							
II	13	38.5 [29.55–48.3]	61.5 [51.7–70.45]	.020	69.2 [59.56–77.42]	30.8 [22.58–40.44]	.143
III	26	53.9 [44.17–63.34]	46.1 [36.66–55.83]		76.9 [67.68–84.13]	23.1 [15.87–32.32]	
Tumor grade							
I	2	100 [95.56–100]	0 [0.0–4.441]	NA^a^	100 [95.56–100]	0 [0.0–4.441]	NA^a^
II	4	25 [17.5–34.35]	75 [65.65–82.5]		62.5 [52.7–71.37]	37.5 [28.63–47.3]	
III	34	50 [40.38–59.62]	50 [40.38–59.62]		75 [65.65–82.5]	25 [17.5–34.35]	

rs1050450 C>T (NM_001329455.1).

CI, confidence intervals; *n*, number; NA, not applied.

Because of the limited number of cases with tumor grade I, statistical analysis couldn't be performed.

a No case has the Leu/Leu genotype; therefore this genotype was not included in the statistical analysis.

Allele frequency analysis showed that Pro allele prevails in both stages II and III, with 69.2% and 76.9%, respectively, and significant association was obtained (*p* = .143). Regarding the distribution of cases according to tumor grade, statistical analysis could not be applied due to limited number of cases mainly in grade I.

## 4 DISCUSSION

During the last decades, a great interest was given to *GPX1* as a determinant of cancer risk. Accordingly, the identification of a well‐characterized functional polymorphism named p.Pro198Leu (rs1050450 C>T) in *GPX1* gene, a lot of studies have been conducted to evaluate the association between p.Pro198Leu polymorphism and risk of cancer development. Therefore, a great interest was given to the association between p.Pro198Leu polymorphism and breast cancer risk in various populations and a strong association was reported in Denmark, USA, UK and Poland (Hu & Diamond, 2003; Jablonska et al., 2009; Méplan, Dragsted, Ravn‐haren, Tjønneland, & Vogel, 2013; Ravn‐Haren et al., 2006), whereas other studies conducted in UK and USA didn't observe any significant association (Ahn et al., 2005; Cebrian et al., 2006; Cox et al., 2004). A well conducted meta‐analysis performed by Hu et al. (2010) indicated the existence of significant ethnic variation in the GPX1 Pro198Leu polymorphism and breast cancer development and suggested that the polymorphic variant may increase the risk only among Africans. However, this conclusion was based in Afro‐American women. To our best knowledge, only three studies, conducted on African ethnicity descents in Egypt and Morocco, have investigated this polymorphism in bladder cancer (Goerlitz et al., 2011; Hadami et al., 2016) and hepatocellular carcinoma (Ezzikouri et al., 2010), but none on breast cancer.

The corner stone of this case–control study is to assess the GPX1 Pro198Leu polymorphism status in Rwanda and to evaluate the association between this polymorphism and breast cancer development among Rwandan patients. In this study, no association between GPX1 Pro198Leu polymorphism and breast cancer development have been reported between cases and control groups (Pro/Leu vs. Pro/Pro: *p *=* *.5). No significant difference was also found between Leu and Pro alleles between cases and controls (*p *=* *.5). Previous studies highlighted the presence of homozygous Leu/Leu genotypes and showed that Leu allele is strongly associated with breast cancer development suggesting that individuals carrying variant Leu allele (Pro/Leu and Leu/Leu) were associated with an increased cancer risk (Chen et al., 2011; Jablonska et al., 2009; Méplan et al., 2013).

Of particular interest, Méplan et al. (2013) have showed that the significant association of GPX1 GPX1 Pro198Leu polymorphism and breast cancer development has been restricted only to the nonductal cancers. In our study, Invasive ductal carcinoma subtype, prevails (36/41), suggesting a probable association between this polymorphism and the histological subtype, deserving further investigations.

Interestingly, the Leu/Leu genotype was not found among Rwandan patients and controls. Leu/Leu genotype has not been reported in studies conducted in Egypt and Morocco, suggesting that this genotype is rare in Africa. Moreover, it's widely accepted that frequency distribution of alleles vary significantly according to ethnicity. Leu allele was found in 36% of Caucasians, in 33% of African descents, 5% of Japanese and was not yet reported in Chinese subjects (Hu & Diamond, 2003; Ichimura et al., 2004).

When considered separately, sporadic and family cases displayed the same distribution both for genotypes and alleles frequencies, suggesting no association between this polymorphism and the hereditary form of breast cancer. However, an increased risk of breast cancer was observed in individuals who carry both the Leu198Leu genotype of *GPX1* and Ala16Ala genotype of MnSOD which is in favor of a GPX1 and MnSOD interaction responsible of an increased risk of breast cancer development (Cox, Tamimi, & Hunter, 2006). This interaction was confirmed in the study performed in Russia by Ermolenko et al. (2010) showing that combination MnSOD Ala9Val and GPX1 Pro198Leu genotypes were found to have a 1.6 times higher risk of sporadic breast cancer as compared to the control group. Thus, evaluation of association of GPX1 Pro198Leu polymorphism with breast cancer would be more interesting when combined with other common variation in other polymorphic genes encoding for antioxidant defence enzymes, including MnSOD, in modulating individual susceptibility to breast cancer (Cebrian et al., 2006).

In the present study, a strong association was found between GPX1 Pro198Leu polymorphism and clinical stage (*p *=* *.02). Pro/Leu genotype was significantly higher in stage II as compared to stage III where the Pro/Pro genotype prevails. The presence of Leu allele seems to have a protective effect against breast cancer development and progression. This finding is supported by a recent study conducted in Poland showing that carrying Leu variant was associated with a significant 40% decrease in the breast cancer risk, suggesting a protective effect of *GPX1* Leu variant (Jaworska et al., 2013). Similar protective effect of *GPX1* Leu variant has been found also in the case of lung and laryngeal cancers (Jaworska et al., 2013). Leu allele is associated with a reduced activity of ROS scavenging activity, and the unexpectedly observed protective effect of Leu allele may be explained by the fact that patients carrying Leu allele have better prognosis after cancer treatment as most of the therapies (immunotherapy, chemotherapy, radiotherapy) are based on ROS generation (Zhao, Liang, Grossman, & Wu, 2005). Association between GPX1 Pro198Leu polymorphism and tumor grade was not applicable because of the very limited number of cases in grades I and II.

Overall, this study is very informative giving evidence of the absence of association between p.Pro198Leu polymorphism and breast cancer development in Rwanda and highlighting the rarity of homozygous Leu/Leu genotype in Rwandan people. However, the main limitation of this study is the small sample size making difficult to assess a clear association between such polymorphism and clinicopathological features.

The results of this preliminary study showed that 198Leu genotype is rare in Rwanda and suggest that GPX1 Pro198Leu polymorphism is not a risk factor for breast cancer development. However large scale investigation in Rwandan and other African patients are needed to draw consistent information on the implication of this polymorphism in breast cancer in African patients.

## CONFLICT OF INTEREST

The authors declare no conflict of interest.
